# Exceptional-Point-Enhanced Chiral Spoof Localized Surface Plasmon Resonator for Sub-Microliter Glucose Microwave Biosensing

**DOI:** 10.3390/bios16080411

**Published:** 2026-07-30

**Authors:** Zengxiang Wang, Wenfei Mo, Zhaoyang Wang, Cuizhen Sun, Xia Xiao, Xiaojun Huang

**Affiliations:** 1 College of Communication and Information Engineering, Xi’an University of Science and Technology, Xi’an 710054, China; 25207223080@stu.xust.edu.cn (W.M.); scz@xust.edu.cn (C.S.); hxj@xust.edu.cn (X.H.); 2 School of Microelectronics, State Key Laboratory of Advanced Materials for Intelligent Sensing, Tianjin Key Laboratory of Imaging and Sensing Microelectronic Technology, Tianjin University, Tianjin 300072, China; zhaoywang@zzu.edu.cn (Z.W.); xiaxiao@tju.edu.cn (X.X.); 3 College of Energy and Mining Engineering, Xi’an University of Science and Technology, Xi’an 710054, China; 4 School of Information Engineering, Zhengzhou University, Zhengzhou 450001, China

**Keywords:** biosensor, glucose detection, exceptional points, spoof localized surface plasmon (SLSP), deep subwavelength

## Abstract

Microwave resonance sensors are promising for dielectric characterization and biochemical detection. However, their sensing performance is constrained by the relatively long wavelength at microwave frequencies and the limited electromagnetic interaction with small-volume samples. In this paper, an exceptional-point (EP)-enhanced chiral spoof localized surface plasmon (SLSP) resonator is proposed for high sensitivity microwave biosensing with sub-microliter sample volumes. By rotating the chiral resonator relative to the microstrip feeding line, two EPs are obtained at α = 49° and α = 210°. The simulated results show that the EP states generate larger frequency splitting than the reference state under both dielectric-constant variation and detection-limit evaluation, confirming the squareroot response to weak perturbations. Experiments further validate the sensing performance using low-loss dielectric materials and high-loss glucose solutions. For dielectric samples with relative permittivities from 2 to 6.15, the frequency splitting increases from 0.287 GHz to 0.359 GHz. For glucose solutions, the frequency splitting decreases from 1.506 GHz to 1.372 GHz as the glucose amount increases from 2.78 nmol to 27.8 nmol. The proposed sensor requires only about 0.5 μL of sample volume and shows higher sensitivity than reported microwave glucose sensors. These results demonstrate that the EP-enhanced chiral SLSP resonator provides a compact and sensitive platform for trace-volume biochemical detection and integrated microwave biosensing.

## 1. Introduction

Electromagnetic resonance sensors are widely used for dielectric constant measurement [1,2,3], in which the dielectric constant of the sample is characterized by resonance frequency shift. It also allows for non-invasive, real-time, and label-free detection [4]. Compared with most traditional methods, resonance sensing usually needs a simpler setup and causes less destructive to the sample. Many types of resonators have been developed for this purpose. Typical examples include radio-frequency resonators [5], optical resonators [6], and microwave resonators [7]. Among them, microwave resonators are especially attractive for biomedical sensing, in vitro diagnosis, and trace liquid analysis. They are compact, low-cost, and easy to integrate with planar circuits. They also use non-ionizing electromagnetic waves and respond strongly to dielectric changes in biological samples [8,9,10].

Planar microwave sensors have been developed in various forms. Common structures include split-ring resonators [11,12,13], spiral resonators [14,15], and interdigital resonators [16,17]. These sensors have shown considerable potential in material testing, liquid sensing, and biomedical detection. However, the long wavelength of microwaves still sets a major limit. It makes the sensing region relatively large, and also weakens the interaction between the resonator and a small amount of sample. Therefore, traditional planar microwave sensors often require a large sample volume and show a limited detection limit. This problem becomes more serious in biomedical sensing, where the available sample volume is typically limited. Microwave glucose sensors have been widely investigated using split-ring resonators, complementary resonators, interdigital structures, microstrip resonators, and fluidic sensing platforms. These sensors are attractive because they allow label-free detection, simple electrical readout, low-cost fabrication, and integration with planar microwave circuits. They can also detect dielectric changes in liquid samples without optical labeling or chemical modification. However, the dielectric contrast introduced by glucose solutions is usually weak, especially at low concentrations. In addition, aqueous glucose samples introduce considerable dielectric loss, which broadens the resonance and reduces the measurable frequency shift. Many reported microwave glucose sensors therefore require microliter- to hundreds-of-microliter sample volumes to obtain a reliable response. These limitations remain important challenges for trace-volume glucose detection.

Spoof localized surface plasmons (SLSP) provide an effective approach for overcoming the size limitation imposed by the long operating wavelength of microwave devices. By compressing electromagnetic energy into deep-subwavelength regions, SLSPs generate highly localized electric fields and substantially enhance the interaction between the resonator and the material under test (MUT). This capability has made SLSP resonators an important platform for compact and high-sensitivity microwave sensing. This strong field confinement increases the overlap between the resonator and the sample. It also improves the sensing response when the sample volume is small. The concept of SLSPs was first introduced by Pendry et al. in 2012 [18]. Shen and Cui subsequently demonstrated this concept experimentally using ultrathin metallic patterns fabricated on printed circuit boards [19]. These reports made SLSP structures practical for planar microwave circuits. Therefore, SLSP resonators have been widely used in compact microwave sensing. SLSP resonators can compress the effective wavelength to deep-subwavelength, even below λ_0_/100. This feature has enabled microliter-level solution sensing and small-volume dielectric testing. Even so, most reported SLSP sensors still work as conventional Hermitian resonators. Their frequency response is usually linear with respect to dielectric perturbation. This linear response is stable and easy to use. However, it also limits the signal change when the perturbation is very weak. This problem becomes important for sub-microliter liquid sensing. In that case, the sample volume is very small, and the induced dielectric change is weak. As a result, field confinement alone is not enough to further improve the detection limit. A new sensing mechanism is needed to turn weak perturbations into larger and more readable frequency changes.

Exceptional points (EP) offer another way to improve sensing sensitivity [20,21,22,23,24]. An EP appears in a non-Hermitian resonant system when two modes merge into one state. At the EP state, the system becomes very sensitive to a small external change. When a sample or a tiny scatterer is placed near the resonator, the EP state is broken. The single resonance then splits into two resonances. This splitting follows a square-root response relation with the perturbation strength. Because of this response, a weak perturbation can produce a larger frequency change than in a normal resonator. EP-based sensing was first shown in optical microcavities. It allowed very small particles to be detected. Later, this idea was extended to microwave, acoustic, and other wave systems. In the field of microwave sensing, it is often combined with SLSP structures to realize the detection of deep-subwavelength targets. However, microwave EP sensors still have several practical problems. Many reported microwave EPs need extra scatterers to tune the system into the EP state [25]. These scatterers can introduce additional loss. They can also reduce the quality factor of the resonator. Therefore, it remains a great challenge to achieve EP states in compact SLSP resonators. This goal is especially important for trace-volume biochemical sensing, where both high sensitivity and small device size are needed.

For sensing applications, chirality and EPs play different but connected roles. The chiral SLSP geometry concentrates the electric field near the patterned slot and increases the overlap between the resonator field and the sample. It also breaks the mirror symmetry of the resonator, allowing for asymmetric coupling between two quadrupole-like modes. This asymmetric coupling provides a way to tune the system toward a non-Hermitian EP. Once the sensor operates near the EP, a weak dielectric perturbation can split the coalesced mode into two resonances. The resulting frequency splitting follows a square-root dependence on the perturbation strength, which is easier to resolve than the linear frequency shift in a conventional resonator when the sample volume is very small. Therefore, chirality mainly provides field localization and modal-control capability, while the EP provides a sensitive frequency-splitting readout. This combined mechanism is especially suitable for microwave SLSP sensors, where localized fields and EP-induced splitting can work together to improve trace-volume biochemical sensing.

In this paper, an exceptional-point-enhanced chiral SLSP resonator is proposed for high-sensitivity microwave biosensing with sub-microliter samples. The proposed resonator uses a chiral slot structure to break the symmetry of a conventional annular SLSP resonator. This design forms a non-Hermitian coupled system based on two quadrupole-like modes. By rotating the chiral resonator relative to the microstrip feeding line, two EP states are realized without using additional scatterers. At the EP states, the resonator exhibits strong field confinement and a square root frequency-splitting response to weak dielectric perturbations. Full-wave simulations are carried out to verify the sensing enhancement under dielectric-constant variation and MUT-size-dependent perturbation. Experiments are further performed using low-loss dielectric materials and high-loss glucose solutions.

## 2. Theoretical Framework and Realization of the Exceptional Point

**Figure 1:**
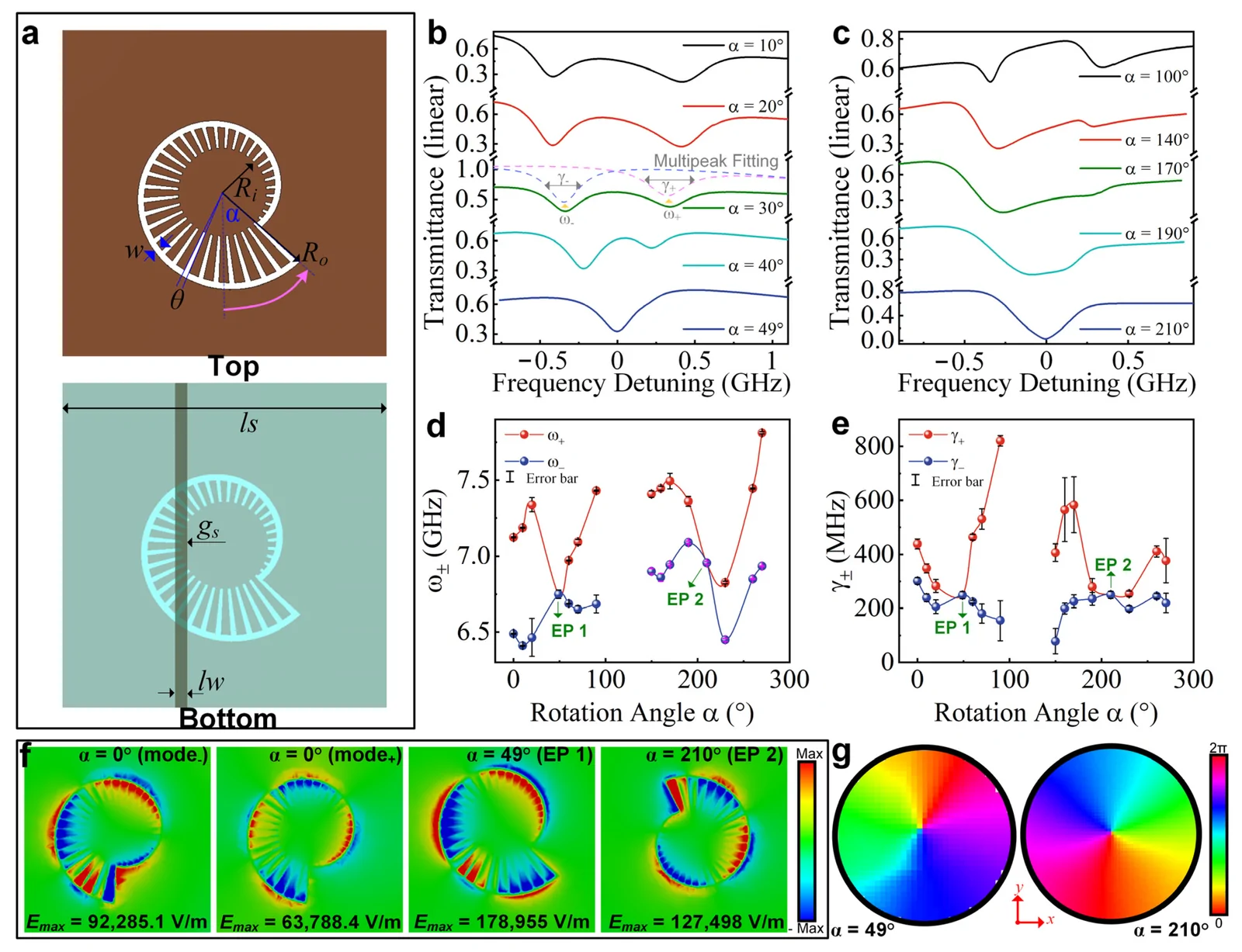
Formation and characterization of exceptional-point (EP) state. (**a**) Geometry of the introduced chiral spoof localized surface plasmon (SLSP) resonator and the corresponding microstrip feeding structure. (**b**) Evolution of the measured transmission spectra as a function of the rotation angle α. (**c**) Schematic illustration of the EP formation process by tuning the relative orientation between the chiral SLSP resonator and the microstrip line. (**d**) Real parts of the complex eigenfrequencies extracted from the transmission spectra. (**e**) Imaginary parts of the complex eigenfrequencies extracted from the transmission spectra. The error bars in (**d**,**e**) represent the standard errors obtained from double-peak Lorentzian fitting. The simultaneous coalescence of the real and imaginary eigenfrequencies identifies the EP states at α = 49° and α = 210°. (**f**) Electric-field distributions of the quadrupolar modes for the reference state (α = 0°) and the two EP states. (**g**) Corresponding phase distributions showing the vortex characteristics of the DP and EP modes.

The chiral SLSP structure is realized by etching a chiral slot pattern on the ground plane and exciting it through a microstrip feed line, as illustrated in Figure 1a. The slot resonator supports LSP-like resonances in the microwave regime, in which the electromagnetic energy is strongly confined around the patterned slot edges. The geometric parameters are designed as *R_i_* = 4.8 mm, *R*_o_ = 12 mm, *w* = 0.5 mm, and θ = 3.6°. The dielectric substrate is the F4B substrate with a thickness of 0.5 mm, a dielectric constant of 2.65, and a loss tangent of 0.001. Compared to conventional symmetric SLSP resonators, the proposed SLSP introduces intrinsic geometric chirality by constraining the inner boundary into an Archimedean spiral profile following x=(a+bt)cos(t) and y=(a+bt)sin(t), where *a* = 2, *b* = 0.9, and $t\in \left[0,6.3\right]$. Herein, the parameter *a* determines the initial position of the spiral and the longest tooth length, and the parameter *b* controls the chiral level of SLSP, corresponding to the tooth-length difference Δ*l* between the longest and shortest unit. In order to achieve EP state in microstrip circuits, chiral SLSP is excited by bottom coupled microstrip lines. As shown in the illustration of Figure 1a, the microstrip line width is *l_w_* = 1.32 mm and its impedance is 50 Ω. To further construct asymmetry, the microstrip line is offset from the center position by *g_s_* = 4.14 mm. Such geometric asymmetry breaks the modal degeneracy of the conventional SLSP resonator and induces strong non-Hermitian coupling between the intrinsic eigenmodes.

Figure 1b,c showed the tuning process of EP states by rotating the chiral SLSP with respect to the microstrip line. Two EPs (EP1 and EP2) are observed at rotation angles of α = 49° and α = 210°, respectively. The two EP angles are associated with the relative alignment between the chiral slot field distribution and the offset microstrip excitation. During rotation, the feeding line samples different parts of the chiral quadrupolar field. This changes the external coupling strength and radiation loss of the two hybridized modes. At α = 49° and α = 210°, the coherent coupling and loss imbalance reach the condition required for non-Hermitian mode coalescence. Away from these orientations, the two modes either have finite frequency detuning or finite damping detuning, so the complex eigenfrequencies do not merge. Thus, the two observed EPs are not arbitrary frequency crossings, but two angular solutions of the non-Hermitian EP condition for the present chiral and offset-fed geometry. The corresponding evolutions of the real and imaginary parts of the complex eigenfrequencies are shown in Figure 1d,e, verifying the simultaneous coalescence of both eigenvalues and eigenvectors at these two angles. The complex resonance frequencies in Figure 1d,e were extracted from the simulated *S*_21_ spectra using double-peak Lorentzian fitting. The error bars represent the standard errors of the fitted resonance frequencies and linewidths, estimated from the fitting covariance matrix. Some error bars are smaller than the marker size, while larger error bars occur when the two resonances overlap or when the linewidth becomes broad. It should also be noted that the apparent evolution of the real and imaginary parts depends on the projection of the complex eigenfrequency trajectories. An EP is a branch point in the complex-frequency plane. When the two complex eigenfrequency branches are plotted separately in terms of their real and imaginary parts as functions of a single real tuning parameter, the projected curves may show crossing or anti-crossing depending on the local direction of the trajectory. By contrast, the case of α = 0° is selected as the reference diabolic point (DP) state. In contrast to a conventional symmetric annular slot resonator, the proposed chiral slot breaks the in-plane rotational symmetry and modifies the modal distribution of the supported resonances. As shown in Figure 1f, the symmetric slot resonator supports two nearly degenerate quadrupole-like eigenmodes with different spatial orientations. After introducing the chiral opening, the two quadrupole modes become non-degenerate and exhibit strongly asymmetric field distributions. This behavior provides the physical basis for constructing an EP. Within the symmetric SLSP configuration, two quadrupole modes are mainly protected by geometric symmetry and remain approximately orthogonal. Such a degeneracy is analogous to a conventional DP. In the chiral resonator, however, the broken symmetry lifts the original degeneracy and produces two coupled, non-degenerate quadrupole modes. The field distributions in Figure 1f show that the chiral slot introduces strong field localization near the opening and curved slot edges, which enhances both intermodal coupling and external coupling to the microstrip line. Compared with the DP state, the EP states exhibit significantly enhanced electric-field confinement. For the DP state, the maximum electric fields of mode_−_ and mode_+_ are approximately 92,285.1 V/m and 63,788.4 V/m, respectively. In contrast, the maximum electric fields of the two EP states increase to 178,955 V/m and 127,498 V/m. It can be seen that the maximum electric field of the EP state almost doubled. Since the sensing sensitivity is proportional to the localized electric-field intensity, the EP states provide much stronger field localization and sensing enhancement than the DP state.

Furthermore, the phase distributions of the EP states shown in Figure 1g reveal a clear first-order vortex feature with a continuous 2π phase winding around the center. Unlike the DP state, where the vortex field originates from the superposition of two orthogonal degenerate modes, the EP vortex mode originates from the coalescence of two non-Hermitian eigenmodes. Therefore, although both EP and DP states exhibit similar vortex-like phase profiles, their physical mechanisms are fundamentally different. To further distinguish the vortex mechanisms of the DP-like reference state and the EP states, the phase topology was quantitatively analyzed using the complex *E_z_* field. It can be written as $E_{x}(x,y)=\left|E_{x}(x,y)\right|e^{j\phi (x,y)}$, where *ϕ*(*x*, *y*) is the local phase. The topological charge was calculated from the phase winding around a closed contour enclosing the vortex core, $q=\oint_{C}\nabla \phi \cdot dl$. For both the DP-like state and the EP states, the phase changes continuously by approximately 2π along the closed contour, corresponding to a first-order vortex with q≈1. Although the topological charge is similar, the modal origins are different. In the symmetric resonator, the vortex field is produced by the superposition of two nearly degenerate orthogonal quadrupole modes with a phase difference close to π/2. In the chiral resonator, the vortex field is associated with the coalesced eigenstate at the EP, where the two non-Hermitian eigenmodes merge in the complex-frequency plane. Therefore, the DP-like vortex is a Hermitian degeneracy-induced vortex, while the EP vortex is a non-Hermitian coalescence-induced vortex.

To describe the non-Hermitian coupling mechanism, the two intrinsic quadrupolar eigenmodes inside the chiral SLSP structure are represented by complex eigenfrequencies ω*_i_* + *j*γ*_i_*, where ω*_i_* denotes the resonant frequency and γ*_i_* denotes the modal damping rate. The modal order does not change the form of the two-mode Hamiltonian, but it determines the effective parameters entering the Hamiltonian. The coupled non-Hermitian system can therefore be expressed using the effective Hamiltonian [26,27].

(1) $$\hat{H}=\left(\begin{array}{cc} \omega_{1}+j\gamma_{1} & \kappa \\ \kappa & \omega_{2}+j\gamma_{2} \end{array}\right)$$

where ω_1_ and ω_2_ are the resonance frequencies of the two uncoupled quadrupole modes, γ_1_ and γ_2_ are their damping rates, and κ is the effective coupling coefficient between them. The damping rates include both intrinsic losses and external losses induced by the microstrip feeding structure. By introducing the average frequency and damping rate, $\omega_{0}=\left(\omega_{1}+\omega_{2}\right)/2$, $\gamma_{0}=\left(\gamma_{1}+\gamma_{2}\right)/2$, and the frequency and damping detunings, $\Delta \omega =\left(\omega_{1}-\omega_{2}\right)/2$, $\Delta \gamma =\left(\gamma_{1}-\gamma_{2}\right)/2$. Therefore, the Hamiltonian can be rewritten as

(2) $$\hat{H}=\left(\begin{array}{cc} \omega_{0}+j\gamma_{0} & 0\\ 0 & \omega_{0}+j\gamma_{0} \end{array}\right)+\left(\begin{array}{cc} \Delta \omega +j\Delta \gamma & \kappa \\ \kappa & -\Delta \omega -j\Delta \gamma \end{array}\right)$$

The complex eigenfrequencies of the coupled system are then given by

(3) $$\Omega_{\pm }=\omega_{0}+j\gamma_{0}\pm \sqrt{\kappa^{2}+{\left(\Delta \omega +j\Delta \gamma \right)}^{2}}$$

where $\Omega_{\pm }=\omega_{\pm }+\gamma_{\pm }$, ω_±_ represent the real resonance frequencies of the two hybridized modes, while γ_±_ denote their damping rates. The two branches Ω_+_ and Ω_−_ correspond to the two resonant features observed in the transmission spectra.

In the proposed structure, the rotation angle α of the chiral slot resonator serves as the tuning parameter. When the chiral slot is rotated with respect to the fixed microstrip line, the spatial overlap between the localized quadrupole fields and the feeding line changes. As a result, the coupling coefficient κ, the frequency detuning Δω, and the damping detuning Δγ are simultaneously modulated,

(4) $$\left\{\begin{array}{c} \kappa =\kappa (\alpha )\\ \Delta \omega =\Delta \omega (\alpha )\\ \Delta \gamma =\Delta \gamma (\alpha ) \end{array}\right.$$

Therefore, varying α drives the system through different non-Hermitian coupling regimes. As shown in Figure 1b,c, the transmission spectra evolve with the rotation angle α, and the two resonance features gradually approach, coalesce, and split again. The extracted resonance frequencies and damping rates are plotted in Figure 1d,e, where two EP candidates, denoted as EP1 and EP2, can be identified from the simultaneous coalescence of the real and imaginary parts of the complex resonance frequencies.

The EP condition is reached when the square-root response term in the eigenfrequency expression vanishes:

(5) $$\kappa^{2}+{\left(\Delta \omega +j\Delta \gamma \right)}^{2}=0$$

Under this condition, the two complex eigenfrequencies coalesce:

(6) $$\Omega_{+}=\Omega_{-}$$

Which requires simultaneous merging of the real and imaginary parts, $\omega_{+}=\omega_{-}$ and $\gamma_{+}=\gamma_{-}$. It should be noted that not every spectral degeneracy corresponds to an exceptional point. In a Hermitian or conventional diabolic degeneracy, two eigenvalues may become degenerate while the corresponding eigenvectors remain independent and orthogonal. In contrast, an EP in a non-Hermitian system requires the coalescence of both the eigenvalues and the eigenvectors. In the present chiral SLSP resonator, non-Hermiticity arises from dielectric loss, conductor loss, radiation leakage, and asymmetric external coupling to the microstrip feeding line. These effects produce different damping rates and unequal coupling conditions for the two quadrupole-like modes. Therefore, the system is described by a non-Hermitian effective Hamiltonian. The EPs are identified from the simultaneous coalescence of the extracted resonance frequencies and damping rates, together with the defective-mode behavior predicted by the effective Hamiltonian. Thus, the observation of a single dip in the transmission spectrum alone is not sufficient to confirm an EP. A true EP requires the coalescence of both resonance frequencies and damping rates, together with the coalescence of the corresponding eigenstates. In this work, the EP states are identified by extracting ω± and γ± from the transmission spectra using a multipeak fitting method.

For rotation angles away from the EP, the two hybridized quadrupole modes appear as two overlapping resonances in the transmission spectrum. Because the two resonances are partially overlapped and superimposed on a slowly varying background, their resonance parameters cannot be reliably obtained by directly reading the spectral minima. Therefore, the transmission spectra are fitted using two Lorentzian resonances. The Lorentzian model is used for local parameter extraction, not as a claim that all resonances of the open microwave structure are exactly Lorentzian. The transmission amplitude can be expressed as

(7) $$\left|T(f)\right|=B(f)-\frac{A_{-}}{1+4{\left(\frac{f-\omega_{-}}{\gamma_{-}}\right)}^{2}}-\frac{A_{+}}{1+4{\left(\frac{f-\omega_{+}}{\gamma_{+}}\right)}^{2}}$$

where *B*(*f*) is a slowly varying background, A_±_ are the resonance amplitudes, ω_±_ are the fitted resonance frequencies, and γ_±_ are the full widths at half maximum. At the EP angle, the two resonances merge and can be described by a single effective resonance satisfying.

(8) $$\left\{\begin{array}{c} \omega_{EP}=\omega_{+}=\omega_{-}\\ \gamma_{EP}=\gamma_{+}=\gamma_{-} \end{array}\right.$$

This fitting procedure allows the complex resonance frequencies to be tracked as a function of the rotation angle.

The symmetric annular slot supports two quadrupole eigenmodes that are nearly degenerate due to rotational symmetry. In this configuration, the modal fields remain orthogonal and the eigenfrequencies are separated only by a small residual splitting determined by fabrication asymmetry and radiation leakage. When the geometry is modified to a chiral slot, the symmetry constraint is removed. The modal basis is no longer preserved. The two quadrupole-like states become coupled through radiation and loss channels, leading to a non-Hermitian eigenvalue problem. In this condition, the eigenvalues and eigenvectors evolve in the complex-frequency plane and converge at a EP. At this point, both the eigenfrequency and the corresponding mode vector satisfy a defective condition.

External dielectric loading or the introduction of a subwavelength scatterer perturbs the system away from this coalescence. The perturbation modifies both the diagonal and off-diagonal terms of the effective Hamiltonian, which results in a splitting of the previously merged eigenvalues. For a second-order degeneracy, the eigenvalue separation follows a square-root response $\Delta \omega \propto \sqrt{\delta }$ scaling with respect to the perturbation parameter δ. In the weak-perturbation limit, this dependence differs from the linear eigenvalue response observed in non-degenerate systems. The field distribution is concentrated near the slot boundary where the modal overlap is maximum. In this region, the perturbation couples efficiently to both eigenstates and governs the magnitude of the spectral splitting.

## 3. EP-Enhanced Sensing Performance

**Figure 2:**
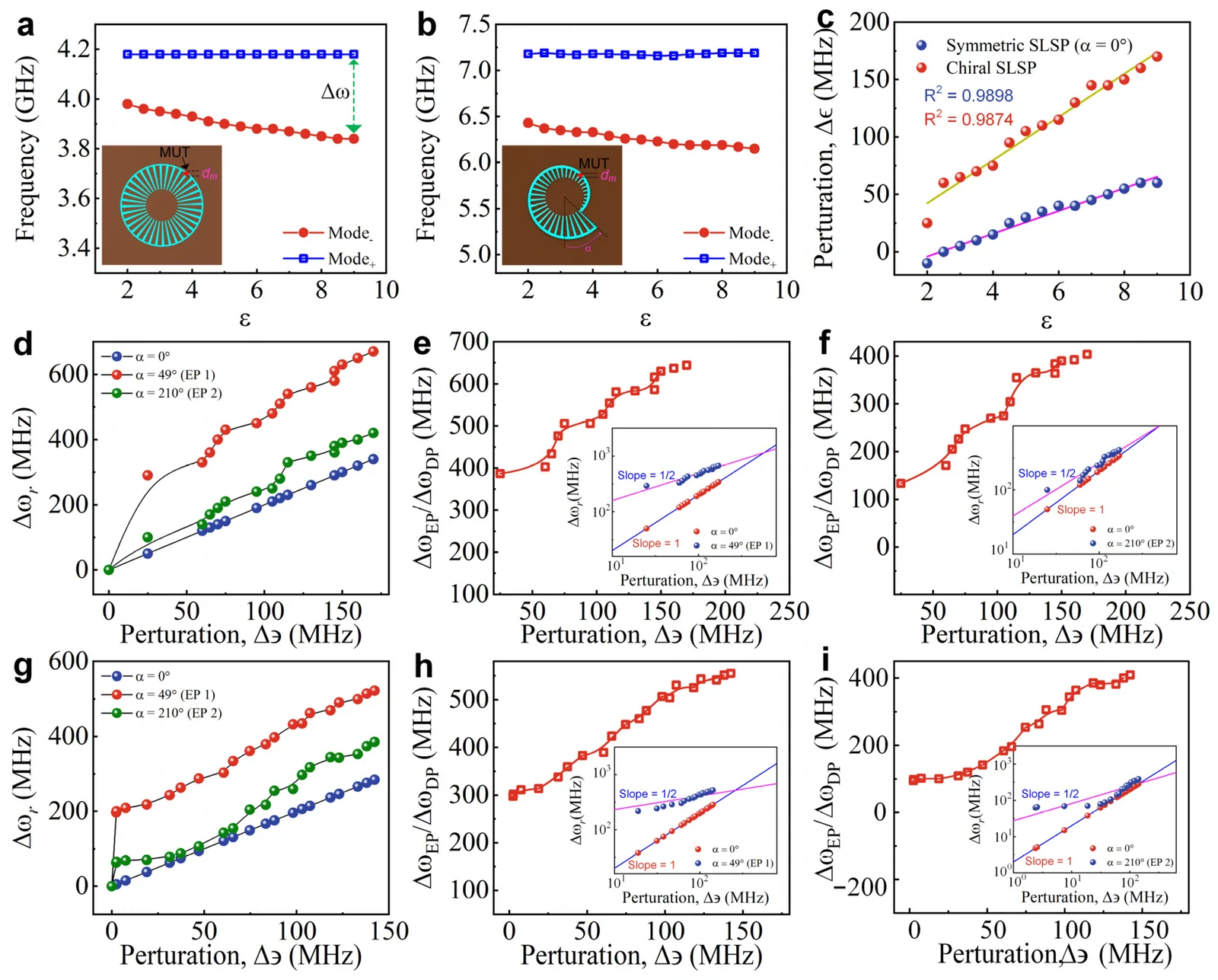
EP-enhanced sensing performance of the SLSP resonators. (**a**) Resonant frequencies of the two split modes when the MUT is loaded on the symmetric SLSP resonator. The inset shows the corresponding MUT loading configuration. (**b**) Resonant frequencies of the two split modes when the MUT is loaded on the chiral SLSP resonator. The inset shows the rotated chiral structure and the MUT position. (**c**) Perturbation-induced frequency splitting as a function of the dielectric constant for the symmetric and chiral SLSP resonators, together with the corresponding linear fitting results. (**d**–**f**) Lossy dielectric sensing performance. (**d**) Relative frequency splitting Δω*_r_* as a function of perturbation strength for α = 0°, α = 49° and α = 210°. (**e**,**f**) Enhancement factors of EP1 and EP2 compared with the α = 0° reference state. Insets show the log–log analysis of Δω*_r_* versus perturbation strength. (**g**–**i**) Detection-limit evaluation with varying MUT size. (**g**) Relative frequency splitting as a function of perturbation strength. (**h**,**i**) Enhancement factors of EP1 and EP2 compared with the reference state. Insets show the corresponding log–log scaling behavior.

To verify the sensing enhancement associated with the EP, full-wave simulations were performed under two representative perturbation scenarios, including to lossy dielectric sensing and detection-limit evaluation. The material under test (MUT) was placed near the edge of the top-layer slit of the SLSP resonator, where the localized electric field is strongly confined. The corresponding loading configurations for the symmetric and chiral SLSP resonators are shown in the insets of Figure 2a and Figure 2b, respectively. Electromagnetic simulations were performed using the frequency-domain solver of CST Microwave Studio. The frequency range is 6 to 9 GHz. The solver accuracy was set to 10^−6^. A total of 10,001 frequency sampling points were used to ensure accurate extraction of the resonant frequencies and frequency splitting. An adaptive tetrahedral mesh was employed, with local mesh refinement applied around the chiral slot, microstrip line, and MUT loading region. The mesh adaptation was continued until the change in the S-parameters between two consecutive passes was below 0.02.

For the lossy dielectric sensing case, the diameter *d_m_* and thickness of MUT are 1 mm and 0.5 mm, respectively. The loss tangent was fixed at 0.02, while the relative permittivity ε was varied from 2 to 9. The resonant-frequency evolutions of the two split modes for the symmetric SLSP resonator and chiral SLSP resonator at α = 0° are shown in Figure 2a and Figure 2b, respectively. The symmetric SLSP resonator is used as a reference because its modal splitting under dielectric loading originates from conventional perturbation-induced symmetry breaking. In contrast, the chiral SLSP resonator is tuned to an EP before loading, so the MUT perturbs a non-Hermitian degeneracy rather than an ordinary Hermitian degeneracy.

For both resonators, the modal frequency separation gradually increases with the relative permittivity of the MUT. This result indicates that the loaded dielectric sample produces a controllable perturbation in the coupled SLSP system. The perturbation-induced frequency splitting Δ_ε_, extracted from the two resonant modes, exhibits an approximately linear dependence on the dielectric constant, as shown in Figure 2c. The fitted coefficients of determination are *R*^2^ = 0.9898 for the symmetric SLSP resonator and *R*^2^ = 0.9874 for the chiral SLSP resonator, where *R*^2^ represents the goodness of fit of the linear regression. It confirms good sensing linearity in both cases. Away from the EP, the two modes remain separated and the eigenvalue response is analytic with respect to weak dielectric loading. The frequency shift is mainly determined by the overlap between the dielectric perturbation and the electric-field energy of the unperturbed mode. When the MUT size and loading position are fixed, this overlap varies approximately in proportion to the dielectric contrast of the MUT. Therefore, the frequency splitting of the reference state shows an approximately linear dependence on the perturbation strength.

Compared with the symmetric SLSP resonator, the chiral SLSP resonator produces a much larger perturbation-induced frequency splitting under the same dielectric loading. This improvement can be attributed to the asymmetric geometry of the chiral SLSP structure, which provides stronger local field confinement around the sensing region and enhances the interaction between the MUT and the resonant field.

To further investigate the EP-induced enhancement, the chiral SLSP resonator was tuned to the EP states by rotating the structure. According to the modal analysis in the previous section, two EP states are realized at α = 49° and α = 210°. Their sensing responses were compared with that of the α = 0° reference state. Following the definition commonly used in EP sensing, the perturbation strength Δ϶ is defined as half of the relative frequency splitting of α = 0° case [28], which is used to quantify the effective dielectric disturbance introduced by the MUT. Therefore, a larger Δ϶ therefore represents a stronger interaction between the MUT and the localized SLSP field. The relative frequency splitting is defined as Δω*_r_* = Δω − Δω_0_, where Δω is the frequency splitting after perturbation and Δω_0_ is the initial frequency splitting of the unperturbed state. For the α = 0° reference state, Δω_0_ is approximately 700 MHz, while for the two EP states at α = 49° and α = 210°, Δω_0_ = 0 MHz due to the coalescence of the two resonant modes. As shown in Figure 2d, the two EP states exhibit much larger relative frequency splitting than the α = 0° reference state under the same perturbation strength. In particular, the EP state at α = 49° shows the strongest response among the three cases. The enhancement factors Δω*_EP_*/Δω*_DP_*, defined as the ratio between the EP-induced splitting and the reference-state splitting, are plotted in Figure 2e and Figure 2f for EP1 and EP2, respectively. Both EP states provide clear sensing enhancement over the reference state. The insets of Figure 2e,f show the corresponding log–log plots of Δω*_r_* as a function of perturbation strength. The reference state follows an approximately linear dependence with a slope close to 1, whereas the EP states exhibit a slope close to 1/2. This result verifies the square-root response of a second-order EP, $\Delta \omega_{EP}\propto \sqrt{\Delta \varepsilon }.$ Therefore, the enhanced sensing performance originates from the non-Hermitian degeneracy of the EP rather than only from ordinary field localization.

The detection-limit performance was further evaluated by varying the physical size of the MUT while keeping its dielectric properties unchanged. In this case, the MUT diameter *d_m_* was varied from 0.2 mm to 4 mm. By changing the size of the MUT, the interaction volume between the dielectric perturbation and the localized SLSP field can be controlled, allowing the weak-perturbation detection capability of the EP sensor to be assessed.

The simulated results are shown in Figure 2g–i. Figure 2g compares the relative frequency splitting Δωr for the α = 0° reference state and the two EP states at α = 49° and α = 210°. Similar to the dielectric-sensing case, both EP states produce larger frequency splitting than the reference state. The enhancement is especially pronounced when the perturbation is weak, corresponding to a small MUT size. This indicates that the EP state is particularly advantageous for detecting miniaturized or trace-volume dielectric targets.

Figure 2h and Figure 2i show the enhancement factors for EP1 and EP2, respectively. The enhancement remains significant over a broad perturbation range, further confirming the improved sensing capability of the EP states. The log–log plots in the insets again show that the EP response follows a square root dependence, while the α = 0° reference state approximately follows a linear perturbation response. This behavior is consistent with the theoretical response of a second-order EP and demonstrates that small perturbations can be converted into more pronounced spectral splitting. The theoretical model in Section 2 provides an effective two-mode description of the EP formation. It captures the coupling between two dominant quadrupole-like modes and explains the square root scaling of the frequency splitting near the EP. However, the full-wave simulations in this Section include the complete three-dimensional electromagnetic response of the practical structure, including finite geometry, feeding-line coupling, radiation leakage, material loss, MUT loading, and background transmission. These factors are not fully considered in the simplified Hamiltonian model. The effective Hamiltonian model explains the origin of EP formation and the perturbation-scaling law, whereas the full-wave simulations evaluate the practical electromagnetic response of the implemented sensor.

Overall, the results in Figure 2 demonstrate that the proposed EP-enhanced chiral SLSP resonator provides improved sensing performance in both dielectric-constant variation and sample-size-dependent response. By combining strong near-field confinement with the square root perturbation response of the EP, the proposed structure enables high-sensitivity microwave sensing of weak dielectric perturbations and small-volume targets.

## 4. Experimental Verification of EP-Enhanced Sensing

**Figure 3:**
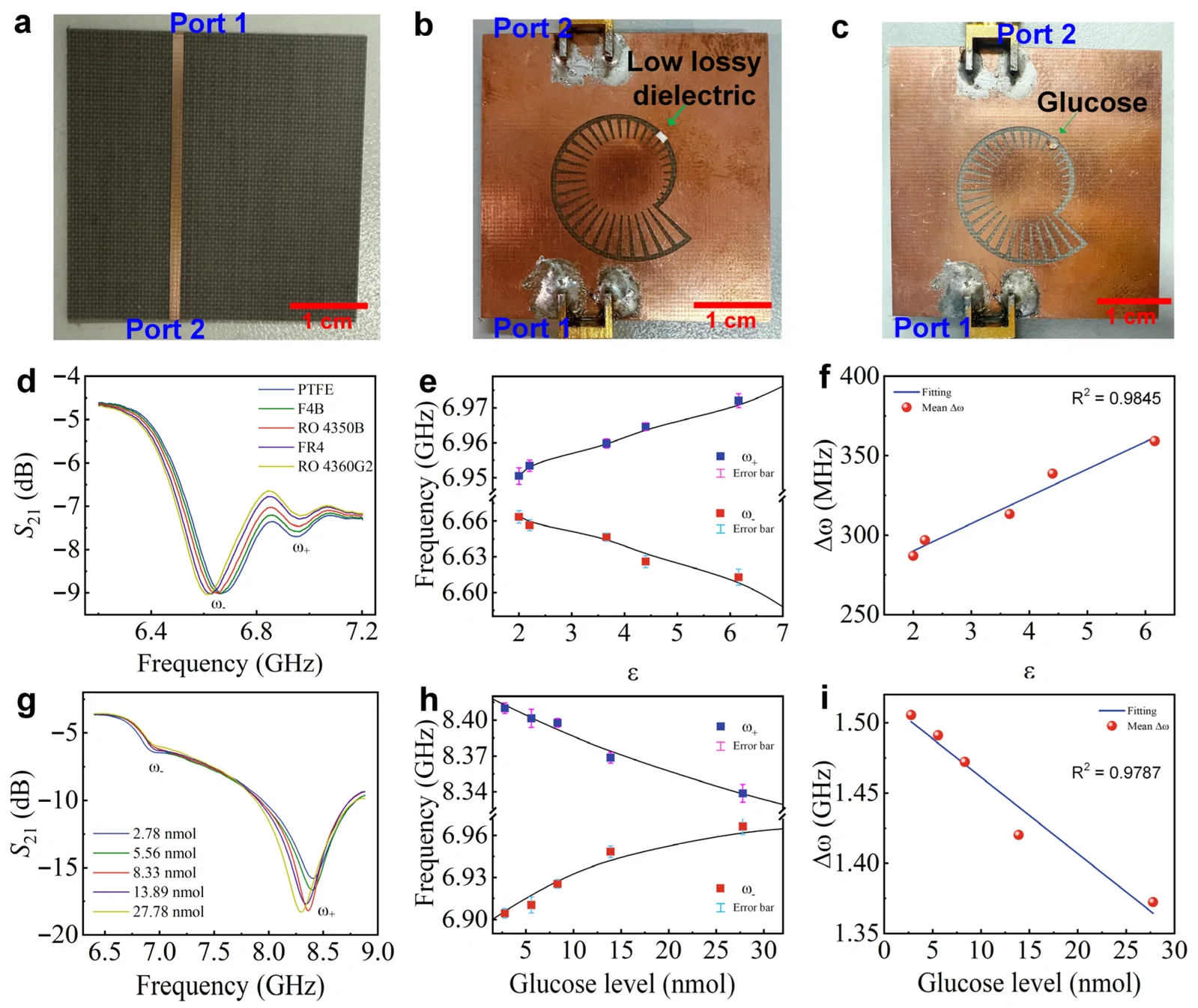
Experiments of EP-enhanced sensing response. (**a**) Fabricated microstrip feeding structure. (**b**) Measurement configuration for low-loss dielectric material sensing. (**c**) Measurement configuration for glucose solution sensing. (**d**) Measured transmission spectra of the sensor loaded with different low-loss dielectric materials. (**e**) Extracted resonance frequencies of the two split modes for low-loss dielectric materials. The symbols represent the mean values from three repeated measurements, and the error bars represent the standard deviations. (**f**) Mean frequency splitting Δω as a function of relative permittivity, with the coefficient of determination *R*^2^ = 0.9845 obtained from linear fitting. (**g**) Measured transmission spectra of glucose solutions with different concentrations. (**h**) Extracted resonance frequencies of the two split modes for glucose solutions. The symbols represent the mean values from three repeated measurements, and the error bars represent the standard deviations. (**i**) Mean frequency splitting Δω as a function of glucose amount, with the coefficient of determination *R*^2^ = 0.9787 obtained from linear fitting.

To verify the sensing capability of the proposed quadrupolar EP sensor, two representative experiment scenarios were investigated. First, low-loss dielectric materials with different relative permittivities were measured to evaluate the dielectric characterization capability. Second, glucose solutions with different concentrations were tested to verify the sensing performance in high-loss biochemical environments. The fabricated prototype and measurement configurations are shown in Figure 3a–c, in which the volume of the tested medium material is approximately 0.5 mm^3^, and the volume of glucose solution is 0.5 uL.

The SLSP resonator was fabricated on a printed circuit board (PCB). The transmission response was measured with a vector network analyzer (VNA), with two SMA connectors used for transmitting and receiving signal. In the measurements, the MUT was placed near the outer edge of the chiral SLSP resonator, corresponding to the strong-field region where the EP state is most sensitive to external perturbations. To evaluate the repeatability of the trace-sample measurements, the complete measurement procedure was repeated three times for both low-loss dielectric sensing and glucose solution sensing. In each repetition, the sample was loaded onto the sensing region, the *S*_21_ spectrum was recorded, and the two resonant frequencies were extracted independently.

### 4.1. Low-Loss Dielectric Material Sensing

Five dielectric materials were used to examine the sensor response under low-loss loading, including polytetrafluoroethylene (PTFE), F4B, Rogers RO4350B, FR4, and Rogers RO4360G2. These materials are widely used microwave substrates with relatively stable and well-characterized dielectric properties. Their relative permittivities cover the range from 2 to 6.15 as listed in Table 1, which allows the sensor response to be examined under controlled dielectric loading. Compared with aqueous samples, these solid substrates introduce weaker dielectric loss and provide repeatable loading conditions. Therefore, they were used first to evaluate the basic dielectric-sensing capability of the proposed EP-enhanced resonator before glucose solution measurements. For each measurement, the substrate was placed on the high-field region near the chiral SLSP resonator, where the EP state is most sensitive to external dielectric perturbations.

Figure 3d shows the measured transmission spectra for different dielectric materials. With increasing dielectric constant, the resonance positions shift more clearly in frequency. The two split resonant modes move in opposite directions under dielectric loading. This behavior corresponds to a stronger perturbation acting on the localized quadrupolar mode. The observed response reflects the change in modal coupling caused by dielectric loading, and the EP degeneracy is lifted under this perturbation condition. In order to reflect the changing trends of ω_−_ and ω_+_ more clearly, the resonant frequencies are extracted separately, as shown in Figure 3e. As the dielectric constant increases, the lower-frequency branch ω_−_ shifts toward lower frequency, while the higher-frequency branch ω_+_ shifts toward higher frequency over the same permittivity range. The frequency splitting is defined as Δω = ω_+_ − ω_−_, and it increases monotonically with permittivity. Figure 3f shows the variation in Δω. The value increases from 0.287 GHz to 0.359 GHz when the relative permittivity changes from 2 to 6.15. This corresponds to a total change of more than 72 MHz. The linear fitting gives R^2^ = 0.9845. The sensor response allows clear discrimination of low-loss dielectric materials with different permittivities.

### 4.2. High-Loss Glucose Solution Sensing

To further evaluate the sensing performance under lossy conditions, glucose solutions with different concentrations were used as MUTs. Compared with solid dielectric materials, glucose solutions exhibit dielectric loss. It provides a more realistic situation for biochemical sensing applications.

Figure 3g shows the measured transmission spectra for glucose amount ranging from 2.78 nmol to 27.78 nmol. With increasing glucose concentration, the resonance frequencies shift within the spectrum. The spectral change remains visible even when the samples introduce strong dielectric loss. The EP-induced mode splitting can still be resolved under these conditions. Different from low-loss dielectric materials, the dielectric constant of glucose solution decreases as the glucose concentration increases [29]. Under this condition, the perturbation to the resonator becomes weaker at higher glucose concentrations. Figure 3h,i show the extracted frequency splitting. The splitting decreases from 1.506 GHz to 1.372 GHz when the glucose amount increases from 2.78 nmol to 27.8 nmol, corresponding to a total change of approximately 134 MHz. The variation in frequency splitting with glucose amount follows a highly linear trend, with R^2^ = 0.9787. This behavior allows quantitative evaluation of glucose concentration even under high-loss sensing conditions.

### 4.3. Comparison and Discussion

The measurements confirm the EP-enhanced sensing response for both low-loss and high-loss samples. The solid dielectric samples show a clear increase in frequency splitting. When the relative permittivity increases from 2 to 6.15, the splitting increases from 0.287 GHz to 0.359 GHz. The total increase is more than 72 MHz. The glucose solutions show a different trend. The effective dielectric constant of the solution decreases as the glucose concentration increases. As a result, the resonance splitting decreases from 1.506 GHz to 1.372 GHz when the glucose amount increases from 2.78 nmol to 27.8 nmol. The total change is about 134 MHz.

To further evaluate the sensing performance, the proposed sensor is compared with previously reported microwave glucose sensors, as summarized in Table 2. Most reported microwave sensors require relatively large sample volumes, typically from several microliters to hundreds of microliters. In contrast, the proposed EP-enhanced SLSP sensor only requires an extremely small sample volume of approximately 0.5 mm^3^, corresponding to about 0.5 µL. Despite the greatly reduced sample volume, the proposed sensor still exhibits much higher sensitivity.

For low-loss dielectric detection, the sensitivity reaches 16.87 MHz, which is several orders of magnitude higher than those reported in previous glucose microwave sensors. For glucose solution detection, the sensitivity is 148.9 × 10^−3^ MHz. This value is lower than that for solid low-loss samples. However, it is still several orders of magnitude higher than the reported microwave glucose sensors. The lower sensitivity in glucose detection is mainly attributed to the high-loss nature of aqueous glucose solutions. Compared with solid dielectric materials, glucose solutions introduce stronger dielectric loss and field dissipation, which weaken the resonant response and reduce the extracted frequency-splitting variation.

The proposed EP resonator still shows a clear frequency-splitting response under high-loss liquid loading. This result shows that the EP-enhanced mechanism can work for both low-loss dielectric samples and biochemical samples. Table 2 shows that the proposed sensor only needs a sub-microliter sample volume. It also gives a much stronger response than conventional microwave sensors. For low-lossy dielectric sensing, the sensitivity improves by about three to six orders of magnitude. For glucose solution sensing, the sensitivity improves by about one to four orders of magnitude. The glucose sensitivity is lower than the solid-sample sensitivity because aqueous samples have strong dielectric loss. Even so, the glucose response is still higher than most reported microwave glucose sensors. These results come from two effects, which are strong SLSP field confinement and the square root response near the exceptional point.

Overall, the proposed EP-enhanced chiral SLSP resonator provides an effective platform for high-sensitivity microwave sensing with extremely small sample volumes. The results demonstrate its potential for compact dielectric characterization, trace-volume biochemical detection, and integrated microwave sensing systems.

It should also be noted that the present glucose measurements were performed using aqueous glucose solutions as proof-of-concept biochemical samples. For practical use, the same PCB-based sensing circuit may be reused after removing the sample and cleaning the sensing region. For solid dielectric measurements, the MUT can be replaced directly. For liquid measurements, however, residual droplets and surface contamination may affect the next measurement. Therefore, the sensing area should be cleaned and dried between tests, and repeated-loading experiments are needed to evaluate the repeatability and long-term stability of the device.

Real biological samples will introduce more complicated dielectric environments than pure glucose solutions. Biological fluids such as serum, sweat, and interstitial fluid contain salts, proteins, metabolites, and other interfering components. These components can change both the effective dielectric constant and dielectric loss of the sample. Some additives may also introduce additional electromagnetic or magnetic responses. Thus, the response measured from pure glucose solutions cannot be directly regarded as the final sensing performance in complex biological media. Previous microwave glucose sensing studies have shown that controlled serum-based mixed samples can be used to evaluate selectivity by comparing the response to glucose with the response to common interferents [30]. Following this strategy, future work will test the present EP-enhanced SLSP sensor using artificial or serum-based mixed samples. Meanwhile, reconfigurable EP-state sensors may be developed by loading tunable capacitors, such as varactor diodes, at selected positions of the chiral SLSP resonator.

**Table 2:** Comparison with previously reported microwave sensors for glucose and dielectric sensing.

	Frequency Range (GHz)	Sample Volume (uL)	Sample	Sensitivity
Ref. [31]	4	200	glucose	10.6 × 10^−6^ MHz
Ref. [32]	1.156	10	glucose	7.6 × 10^−6^ MHz
Ref. [33]	5	40	glucose	5.56 × 10^−6^ MHz
Ref. [34]	0.61–1.75	0–300	glucose	4.73 × 10^−3^ MHz
this work	6–9	≈0.5	low-loss dielectric	17.35 MHz
		0.5	glucose	148.9 × 10^−3^ MHz

## 5. Conclusions

In this paper, an EP-enhanced chiral SLSP resonator for high-sensitivity microwave sensing was proposed and conducted in experimental investigation. The chiral slot structure breaks the symmetry of the conventional annular SLSP resonator and forms a non-Hermitian coupled system. By rotating the resonator relative to the microstrip feeding line, two EP states were obtained at α = 49° and α = 210°. The simulated results confirmed the EP-enhanced sensing mechanism. Compared with the reference state, the EP states produced larger frequency splitting under the same perturbation. The log–log results also showed the square root response near the EP, which explains the higher sensitivity to weak perturbations. These results indicate that the proposed non-Hermitian SLSP resonator can convert small perturbations into more distinguishable frequency changes. Meanwhile, the experiments verified the sensing performance using both low-loss solid dielectrics and high-loss glucose solutions. For solid dielectric samples, the frequency splitting increased by more than 72 MHz when the relative permittivity changed from 2 to 6.15. For glucose solutions, the splitting changed by about 134 MHz when the glucose amount increased from 2.78 nmol to 27.8 nmol. Although the glucose sensitivity was lower than that for solid dielectrics because of the high loss of aqueous samples, it still exceeded most reported microwave glucose sensors. The proposed sensor also required only about 0.5 μL sample volume.

## Figures and Tables

**Table 1:** Key parameters of the three tested materials (MUTs).

Material	*ε_r,mut_*	tan*δ*	*h_mut_* (mm)
PTFE	2	0.0002	0.5
F4B	2.65	0.001	0.5
Rogers RO4350B	3.66	0.0031	0.762
FR4	4.4	0.02	0.5
Rogers RO4360G2	6.15	0.0035	0.635

## Data Availability

The original contributions presented in this study are included in the article. Further inquiries can be directed to the corresponding author.
